# PREVALENCE AND RISK FACTORS OF HYPERKALEMIA AFTER LIVER TRANSPLANTATION

**DOI:** 10.1590/0102-672020180001e1357

**Published:** 2018-06-21

**Authors:** Helem Sena RIBEIRO, Michelle Carvalho OLIVEIRA, Lucilene Rezende ANASTÁCIO, Simone Vasconcelos GENEROSO, Agnaldo Soares LIMA, Maria Isabel CORREIA

**Affiliations:** 1 Postgraduate Program in Applied Sciences for Surgery, Faculty of Medicine;; 2 Department of Nutrition, Schoolof Nursing;; 3 Department of Food Science, Faculty of Pharmacy;; 4 Hospital das Clínicas, Alpha Institute of Gastroenterology, Federal University of Minas Gerais, Belo Horizonte, MG, Brazil.

**Keywords:** Transplants, Allografts, Liver cirrhosis, Potassium., Transplante, Cirrose hepática, Potássio.

## Abstract

**Background:**

There is a lack of data regarding hyperkalemia after liver transplantation.

**Aim:**

To evaluate the prevalence of hyperkalemia after liver transplantation and its associated factors.

**Methods:**

This retrospective cohort study evaluated 147 consecutive post-transplant patients who had at least one year of outpatient medical follow up. The data collection included gender, age, potassium values, urea, creatinine, sodium and medication use at 1, 6 and 12 months after. Hyperkalemia was defined as serum potassium concentrations higher than 5.5 mEq/l.

**Results:**

Hiperkalemia was observed in 18.4%, 17.0% and 6.1% of patients 1, 6 and 12 months after tranplantation, respectively. Older age (p=0.021), low creatinine clearance (p=0.007), increased urea (p=0.010) and hypernatremia (p=0.014) were factors associated with hyperkalemia, as well as the dose of prednisone at six months (p=0.014).

**Conclusion:**

Hyperkalemia was prevalent in less than 20% of patients in the 1^st^ month after liver transplantation and decreased over time. Considering that hyperkalemia does not affect all patients, attention should be paid to the routine potassium intake recommendations, and treatment should be individualized.

## RESUMO

**Racional:**

Existe lacuna na literatura em relação à presença de hipercalemia em pacientes submetidos ao transplante hepático.

**Objetivo:**

Avaliar a prevalência de hipercalemia após o transplante hepático; e avaliar os fatores de risco associados a essa condição.

**Métodos:**

Estudo de coorte retrospectivo, no qual foram avaliados 147 pacientes submetidos ao transplante hepático, com tempo de seguimento de um ano. A coleta de dados compreendeu gênero, idade, valores séricos de potássio, ureia, creatinina, sódio e medicamentos utilizados no tempo de 1, 6 e 12 meses após o transplante. Hipercalemia foi definida como valores de potássio sérico maiores do que 5,5 mEq/l.

**Resultados:**

Hipercalemia foi observada em 18,4%, 17,0% e 6,1% dos pacientes no 1º, 6º e 12º meses após o transplante, consecutivamente. Idade avançada (p=0,021), baixos valores de creatinina sérica (p=0,007), valores aumentados de ureia (p=0,010) e hipernatremia (p=0,014) foram fatores associados à hipercalemia, assim como a dose de prednisona no 6º mês após o transplante (p=0,014).

**Conclusão:**

Hipercalemia esteve presente em menos de 20% dos pacientes no primeiro mês após o transplante hepático, com diminuição da prevalência ao longo do tempo. Considerando que ela não afeta todos os pacientes, o tratamento dietético deve ser individualizado, com especial atenção à recomendação de administração de potássio de forma rotineira.

## INTRODUCTION

Liver transplantation (LTx) is the main treatment for patients with end-stage liver failure and, despite the great benefits of the procedure for survival and quality of life, several important metabolic dysfunctions may occur during the perioperative period and may place these individuals at a higher risk of adverse events. One of these disorders is hyperkalemia, which is defined in adults as serum potassium concentrations higher than 5.5 Eq/l^30^ ^,^ ^15^. Although the most frequent and hazardous hyperkalemia occurs immediately after reperfusion of the newly transplanted liver, the condition may persist throughout time^30^. Progressively, more severe elevations in potassium are responsible for abnormalities in cardiac depolarization and repolarization as well as contractility, also affecting other excitable tissues such as the skeletal muscle^19^.

The causes of hyperkalemia during the early postoperative period are well known^28^, but the reasons related to its maintenance afterwards have not yet been elucidated. Most often, postoperative hyperkalemia seems to be associated with the use of immunosuppressors such as tacrolimus and cyclosporine, which are largely prescribed in the first year after transplantation and are related to hypertension, renal tubule dysfunction, hypercalciuria and acidosis^11^ ^,^ ^14^. Acute and chronic nephrotoxicity are major side effects of both drugs and leads to chronic renal failure in nearly 20% of patients within five years^24^.

The treatment of hyperkalemia is generally based on potassium-binding resins and/or potassium-restricted diets. The dietary restriction of potassium is based on the fact that potassium input to the extracellular compartment comes from both exogenous and endogenous sources. In this regard, nutrients, particularly high-potassium foods, are often restricted in patients undergoing liver transplantation^4^ ^,^ ^8^.

To our knowledge, there are few studies describing the prospective prevalence of hyperkalemia^12^ ^,^ ^13^. This complication has been a long-standing concern, and there is a lack of data describing the prevalence and associated factors of LTx. Addressing this lack of information was the aim of this study.

## METHODS

This is a retrospective cohort study on the prevalence of hyperkalemia and associated factors up to one year after liver transplantation. Patients who underwent LTx between 2001 and 2013 and who regularly attended the outpatient liver transplantation clinic were assessed and had their medical charts reviewed in 2015. The inclusion criteria required complete records, with data encompassing information up to at least one-year post-transplant. Patients who were diagnosed with chronic kidney disease prior to transplantation and patients who underwent dialysis at any time were excluded from the study.

Gender, age and indications for LTx were recorded. The following data were assessed at three different times after the operation (1, 6 and 12 months): medications such as immunosuppressive drugs (tacrolimus, dose and residual dose; cyclosporine and dose; prednisone and dose); antihypertensive drugs (furosemide, spironolactone, propranolol, captopril and enalapril); and serum biochemical markers (potassium, urea, creatinine, sodium). These markers were classified as following: hyperkalemia was defined as serum potassium values higher than 5.5 mEq/l^30^ ^,^ ^15^; increase urea >44 mg/dl; high creatinine >1.5 mg/dl; decreased creatinine clearance - less than 90 ml/min; and hypernatremia >145 mmol/l.

### Statistical analysis

The sample size was calculated using the 2-Sample T-Test, revealing a minimum of 17 patients with hyperkalemia. The sample calculation used a significance level of 0.05 and standard deviation of 5.05 (obtained from the pilot study). The calculation was performed with the Software Mini-Tab Release 14 Statistical Software. The McNemar and Wilcoxon tests were used to identify differences in the prevalence of hyperkalemia, potassium values and the use of drugs throughout the different times after LTx. The Chi-square or Fisher exact test (for categorical variables) or the Student T or Mann-Whitney tests (for numerical variables) were used to evaluate the factors associated with hyperkalemia. Statistical Package for Social Sciences (SPSS) version 20.0 was used for the statistical analysis and p<0.05 was considered significant.

## RESULTS

The medical records of 147 consecutive LTx patients were evaluated. The average age was 50.0±12.7, and there were 69.4% males. The main underlying diseases that indicated transplantation were ethanol cirrhosis 28.6% (n=42), chronic hepatitis C 16.3% (n=24) and cryptogenic cirrhosis 16.3%(n=24), followed by hepatocellular carcinoma 12.9%(n=19), autoimmune hepatitis 6.8%(n=10), primary sclerosing cholangitis 6.1%(n=9), chronic hepatitis B 3.4% (n=5), primary biliary cirrhosis 3.4% (n=5), Wilson’s Disease 2.0% (n=3), hemochromatosis 1.4% (n=2), Caroli’s disease 1.4% (n=2) and fulminant hepatitis 0.7% (n=1) (some patients had more than one disease diagnosis).

Tacrolimus was the most common used immunosuppressor drug. The highest dose of tacrolimus was given in the 1^st^ month, with an average of 16.1±7.1 mg/day, decreasing over time, with a significant difference at 12 months after transplantation. Additionally, the residual dose of the drug decreased over time. The same trend was observed with the doses of cyclosporine, although there was no significant difference between the doses (Table 1). Most patients used prednisone at 30 days after LTx. The dose and the number of individuals using this drug decreased significantly over time.

**TABLE 1:** Characteristics of the use of immunosuppressive drugs, antihypertensive medications and biochemical markers of post-liver transplant patients evaluated from one month up to at least 12 months after the operation (n=147)

Parameters	1 month	6 months	12 months
Immunosuppressive drugs			
Tacrolimus % (n)	97.3 (143)	94.6 (139)	94.6 (139)
Average dose (mg)	16.1±7.1	8.2±4.5*	6.3±3.8*
Residual dose	13.2±5.2	10.0±4.1*	8.2±3.1*
Cyclosporine % (n)	2.7 (4)	5.4 (8)	5.4 (8)
Average dose (mg)	700.0±258.0	387.5±223.2	244.7±166.3
Prednisone % (n)	95.9 (141)	17.7 (26) +	12.9 (19) +
Average dose (mg)	19.9 ± 9.3	6.1 ± 2.7*	5.8 ± 1.9*
Anti hypertensive medications			
Furosemide % (n)	6.8 (10)	4.8 (7)	2.7 (4)
Spironolactone % (n)	0.7 (1)	0.7 (1)	0.0 (0)
Propranolol % (n)	4.1 (6)	2.7 (4)	2.7 (4)
Captopril % (n)	1.4 (2)	2.0 (3)	2.0 (3)
Enalapril% (n)	0.7 (1)	5.4 (8)	4.1 (6)
Biochemical markers			
Hypernatremia% (n)	4.1 (6)	0.0 (0) +	2.0 (3)
Increaseurea% (n)	68.0(100)	58.5 (86)	54.4 (80) +
High creatinine% (n)	17.0 (25)	21.1 (31)	12.2 (18)
Creatinineclearance (ml/min)	71.7±3.4	70.2±30.7	76.5±32.1*
Creatinine clearance < 90 ml/min (n)	79.5 (113)	78.1 (114)	71.4 (105)
Serumpotassium - average (mEq/l)	4.97±0.6	4.88±0.54	4.72±0.49*
Serumpotassium - median (mEq/l) (Minimum - Maximum)	5.0 (3.5-6.6)	4.9 (3.4-6.3)	4.7* (3.1-6.1)

*p<0.05 Wilcoxon and paired sample test; +p<0.05 McNemar test, comparing data from one month after liver transplantation with data from six and 12 months after liver transplantation

There was no significant difference in the use of antihypertensive drugs when comparing 1^st^, 6^th^ and 12^th^ months after transplantation. The number of patients with increased urea was significantly decreased at one year, and creatinine clearance significantly improved. Otherwise, no other marker changed (Table 1).

**FIGURE 1:**
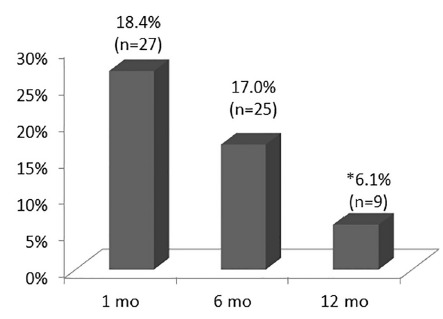
Prevalence of hyperkalemia from one up to 12 months (n=147). Values observed in the 1^st^ month after liver transplantation compared to values observed at six and 12 months (McNemar test: * p<0.05)

Hyperkalemia was observed in 18.4% (n=27) of patients at one month, and 17.0% (n=25) still maintained high potassium levels up to six months after LTx (Figure 1). The prevalence decreased significantly at 12 months compared to the 1^st^ month after transplantation, as did the overall serum potassium values (Table 1).

Increased age at transplantation, lower values of creatinine clearance, increased urea and hypernatremia were risk factors for the presence of hyperkalemia in the 1^st^ month after transplantation. Lower doses of prednisone were associated with hyperkalemia at six months. At 12 months, no risk factor was found to be associated with this condition. The factors associated with hyperkalemia at different times are reported in Table 2. None of the evaluated parameters was associated with hyperkalemia at 12 months.

**TABLE 2:** Risk factors for hyperkalemia by univariate analysis at one and six months after liver transplantation (n=147)

Time afterliver transplantation	Parameters	K<5.5	K=5.5	Odds Ratio; Confidence interval	p
One months	Older age at LTx (years)	49.1±13.1	54.2±9.3		0.021
	Creatinine clearance (ml/min)	73.9±32.5	62.3±15.5		0.007
	Creatinine clearance<90 ml/min	73.3% (n=88/120)	92.6% (n=25/27)	OR= 4.1; CI:0.92-18.5	0.048
	Increase urea	63.3% (n=76/120)	88.9% (n=24/27)	OR= 4.6; CI:1.32-16.27	0.010
	Hypernatremia	16.7% (n=1/120)	83.3% (n=5/27)	OR= 27.0 CI: 3.01-242.79	<0.001
Six months	Prednisone dose (mg)	20.8±9.4	15.88±8.3		0.014

## DISCUSSION

Few studies have addressed the prevalence of hyperkalemia after liver transplantation and its potential associated factors^12^ ^,^ ^13^. In this study, different prevalences of hyperkalemia were observed over time after transplantation, with a significant reduction after 12 months. The small number of studies encompassing the topic and the limitations in comparing the available data because of the different definitions of hyperkalemia as well as the distinct assessed times hamper us in comparing and generalizing our findings. A study by Jain et al. (1999)^13^ reported that 54.0% of patients undergoing liver transplantation presented hyperkalemia (>5.0 mEq/l) in the 1^st^ year, 50% up to three years, and one-third of patients at the 7^th^ year after the operation^13^. In our study, the prevalence of hyperkalemia in the 1^st^ year after transplantation, considering the same cutoff of 5.0 mEq/l, was 25.2% (n=37, data not shown). The differences in prevalence between the two studies may be explained because there was no exclusion of patients with renal disease before LTx in the former, and the cutoffs for hyperkalemia in the two studies are different.

In this study, age at transplantation was a risk factor associated with hyperkalemia in the 1^st^ month after LTx. Age is also associated with decreased kidney function, which may be reflected in high levels of potassium. Renal function remains good until the fourth decade of life but thereafter begins to decline by 10% per decade^29^. As a result, adults older than 40 years, but especially over 70, are particularly susceptible to renal adverse events. Thus, chronic renal failure is also common in the liver transplant population due in large part to hypertension and to age-related changes in renal function^7^. Advancing age brings senescent changes in both renal architecture and function that can disturb potassium homeostasis. Therefore, geriatric patients should be considered to have a higher risk of developing hyperkalemia^25^.

It is important to note that most patients had impaired renal function, especially in the 1^st^ month. Almost 80.0% of them had creatinine clearance lower than 90 ml/min, reflecting impaired kidney function^6^. In addition, patients exhibited hypernatremia and especially increased urea, features that reflect changes in renal function^22^. Renal dysfunction is a common and significant complication of solid organ transplantation, and serum potassium is used as a marker of renal failure^9^. Moreover, in general, the prevalence of patients with creatinine clearance less than 90 ml/min decreased over time, as did hyperkalemia and immunosuppressive doses, in both groups. All these conditions exhibited the highest prevalence rates in the 1^st^ month. This fact raises the whole discussion around the relationship of immunosuppressive drugs and changes in renal function, particularly hyperkalemia. The required use of immunosuppressive drugs is the cornerstone of immunosuppression following many types of solid organ transplantation. Among these drugs, calcineurin inhibitors (CNIs), such as cyclosporine and tacrolimus, have undoubtedly altered the spectrum of causes of hyperkalemia^2^. Several studies have already evaluated the influence of CNIs on renal function after liver transplantation^18,27,23,5 )^and they have long been known to cause electrolyte abnormalities, including hyperkalemia^10^. However, in this study we were not able to show associations between the use or dose of CNI and hyperkalemia.

Low prednisone dose was a factor associated with hyperkalemia at six months after LTx. This association was unexpected, and we have hypothesized it to be related to individual variability in the dose that can lead to adrenal suppression, which is a common complication of systemic corticosteroids^26^. Hyperkalemia is a common symptom of hypoaldosteronism^17^. Aldosterone enhances the key mechanisms involved in the distal secretion of potassium. It binds to the nuclear mineralocorticoid receptor within the distal tubule and the principal cells in the cortical collecting duct (CCD) and activates Na+/K+-ATPase, thereby increasing Na+ and water reabsorption into the blood and the secretion of K+ into the urine. Aldosterone also upregulates the amiloride-sensitive sodium channels in the apical membrane of CCD and stimulates H+ secretion, thereby influencing the acid/base balance, as well as increasing the number of open potassium channels in the luminal membrane^16^.

Potassium input into the extracellular compartment arises from both endogenous and exogenous sources, and therefore food restrictions have been routinely indicated for liver transplant patients to help control hyperkalemia^20^. The major sources of potassium are fruits, vegetables, beans, tubers, and nuts, important foods rich in fibers and antioxidants, which are essential to prevent malnutrition-associated states such as metabolic syndrome, a condition highly prevalent in post-liver transplantation patients^3^ ^,^ ^4^. Furthermore, there is no concise recommendation on the potassium restriction for patients undergoing liver transplantation, and little is known about their real intake. A study evaluating the food intake of 148 patients who underwent liver transplantation, with an average of 3.5 years follow up after transplant, found that none of the individuals had adequate potassium intake: the median was 2.1 g (minimum 0.9 g and maximum 4.1 g)^4 )^whereas the recommended intake is 4.7 mg^21^. The same study demonstrated by multivariate analyses that a low intake of potassium was considered a predictive variable for metabolic syndrome^4^. Moreover, high-quality evidence^1^ shows that increased potassium intake reduces blood pressure in people with hypertension, with no effect on catecholamine concentrations or renal function in healthy adults. Therefore, this important aspect should be better addressed in the long-term care of these. 

 Potassium intake was not evaluated in this study, and we do not know if food consumption might have affected hyperkalemia more than older age and impaired renal function. This lack of information is one limitation of our study. Nevertheless, we do know that our patients do not eat food in the quality and quantities needed to achieve the recommendations of potassium, as we previously showed^4^. Another limitation is specially related to the reliance on medical records that may be subject to lack of information or incomplete data. Nonetheless, our data emphasize the importance of further assessing the consumption of potassium among patients undergoing liver transplantation as well as comparing data from different centers with various immunosuppressant drugs and doses.

## CONCLUSION

Hyperkalemia was observed in less than 20% of patients in the 1^st^ month after liver transplantation, with decreased prevalence over time. However, special attention should be paid to older patients and patients with impaired renal function. Therefore, the individualization of treatment is mandatory, and the generalized restriction of potassium intake is not justified.
